# 

**Published:** 1898-03-12

**Authors:** 


					PUS ^<?i,i^i5TBBBHHBBBiBB??TE?^?andard^Tnft>gnrst punty,"?bancet.\ . " makch izthT^ssT
OADBURY'S *J?c,,jjUkBj^^PBURV8
.bso^?Apure. TT tt
8,
OLL
WHVCH HOSPI TAL
^?- 598- Vol. XXIII. IJftgSUTi Saturday,
March 12th, 1898.
j_ see p?408. J PRICE TWOPENCE
JWOPENCE.

				

